# Classification of Fatigue Phases in Healthy and Diabetic Adults Using Wearable Sensor

**DOI:** 10.3390/s20236897

**Published:** 2020-12-03

**Authors:** Lilia Aljihmani, Oussama Kerdjidj, Yibo Zhu, Ranjana K. Mehta, Madhav Erraguntla, Farzan Sasangohar, Khalid Qaraqe

**Affiliations:** 1 Department of Electrical & Computer Engineering, Texas A & M University at Qatar, Doha 23874, Qatar; oussama.kerdjidj@qatar.tamu.edu (O.K.); khalid.qaraqe@qatar.tamu.edu (K.Q.); 2 Department of Industrial & Systems Engineering, Texas A & M University, College Station, TX 77843, USA; boleynsee@gmail.com (Y.Z.); rmehta@tamu.edu (R.K.M.); merraguntla@tamu.edu (M.E.); sasangohar@tamu.edu (F.S.)

**Keywords:** physiological tremor, fatigue, accelerometer, machine learning, classification

## Abstract

Fatigue is defined as “a loss of force-generating capacity” in a muscle that can intensify tremor. Tremor quantification can facilitate early detection of fatigue onset so that preventative or corrective controls can be taken to minimize work-related injuries and improve the performance of tasks that require high-levels of accuracy. We focused on developing a system that recognizes and classifies voluntary effort and detects phases of fatigue. The experiment was designed to extract and evaluate hand-tremor data during the performance of both rest and effort tasks. The data were collected from the wrist and finger of the participant’s dominant hand. To investigate tremor, time, frequency domain features were extracted from the accelerometer signal for segments of 45 and 90 samples/window. Analysis using advanced signal processing and machine-learning techniques such as decision tree, k-nearest neighbor, support vector machine, and ensemble classifiers were applied to discover models to classify rest and effort tasks and the phases of fatigue. Evaluation of the classifier’s performance was assessed based on various metrics using 5-fold cross-validation. The recognition of rest and effort tasks using an ensemble classifier based on the random subspace and window length of 45 samples was deemed to be the most accurate (96.1%). The highest accuracy (~98%) that distinguished between early and late fatigue phases was achieved using the same classifier and window length.

## 1. Introduction

Fatigue or the “loss of force-generating capacity” in muscle [1] can be divided into peripheral and central components depending on the origin of varying levels of the motor pathway [2,3,4]. Central fatigue results from a lack of oxygen reaching the brain or a side effect of inflammatory processes, sleep disturbances, and neurological disorders such as Parkinson’s disease or multiple sclerosis [2,4]. Peripheral fatigue is a “decline in muscle tension (force) capacity with repeated stimulation” [2]. It may occur because of long workdays [5], short intensive actions [6], repeated actions [7], or due to insufficient recovery [8].

An important manifestation of fatigue is tremor [9,10,11,12], defined as “an involuntary oscillation of any part of the body, around any plan” [13]. Two main types of tremor can be distinguished—physiological and pathological. Physiological tremor is often invisible to the naked eye and intensifies as a reaction to stress, exhaustion/fatigue, hypoglycemia, temperature, stimulants, medication, alcohol, tobacco, drug withdrawal, etc. [5,14]. Pathological tremors (such as parkinsonian, essential, cerebral, and neuropathic tremors) ensue due to central or peripheral nervous system disorders or brain injury [15,16]. This type of tremor may disrupt the daily activities of the individual, especially those involving precision tasks, which may, in turn, negatively impact the quality of life [17]. Fatigue-induced physiological tremor can adversely affect tasks that require fine and precise movements (e.g., surgery [5,18], micropipetting [7], or teleoperation of robots [19]).

Two properties, namely frequency and amplitude of the oscillation are usually employed to quantify tremor. The amplitude of oscillation represents a fixed plane’s displacement of and can be used for distinguishing tremors into fine, medium, or coarse categories [20]. It is often used for monitoring the advancement of disorders such as Parkinson’s disease (PD) [15,21]. Compared to pathological tremors, physiological tremors can be considered an artifact [15] because much smaller amplitudes are typical [16]. Furthermore, the displacement depends on age and varies between 0.4 mm to 1.1 mm for young and older individuals, respectively [22,23].

Tremor frequency has been used to categorize tremors concerning movement or body position into either rest or action tremors, with action tremors further classified as postural or kinetic [24,25]. Rest tremor occurs when no voluntary activity is performed, and the body part is entirely supported against gravity. Postural tremor occurs when the affected body part is maintained in a stationary position that is affected by gravity and vanishes with the muscle release. Kinetic tremor appears during voluntary movements and implementation of target-directed tasks such as writing or squeezing an object [15,17,24]. For rest, postural and kinetic tremors, frequencies typically range between 3–6, 4–12, and 3–10 Hz, respectively [26]. Frequency also varies depending on the tremor location. For example, an elbow’s physiological tremor occurs between 3 Hz and 5 Hz, while on the wrist, the frequency ranges between 8 Hz and 12 Hz. With respect to physiological tremor, exercise-induced fatigue results in explicit changes within the time and frequency features of the tremor output [12]. The most prominent impact was increased peak amplitude of the neurally generated 8–12 Hz frequency component [6,12,27]. Intensification of the physiological tremor with the increment of the amplitude when fatigue was being induced is reported by [6,12,28].

Tremor quantification could facilitate early detection of fatigue onset so that preventative or corrective controls are presented to minimize work-related injuries [29] and imprecise performance of tasks requiring high-level accuracy, as well as other cases where is highly essential to make the right decision quickly (surgeons, physicians, pilots, public transport drivers, etc.). In a previous study, we established that signals obtained by an accelerometer-based tremor detection system could capture both the variability and complexity features of fatigue in diabetes patients [30]. Although fatigue occurs in some chronic diseases, the significance of exhaustion might be more noteworthy in people with diabetes [2]. Fatigue is a classic symptom in persons with type 1 diabetes mellitus (T1DM), especially central fatigue, which affects between 23–42% of adults with T1DM. In [31], slower motor conduction velocity and earlier fatigue onset of T1DM subjects than the control group were reported. In the study, surface electromyographic data collection was implemented under glycemic control.

Because quantitative measurements cannot give unconditional information about tremor type, a more objective evaluation that identifies specific features independent of the movement and body fragment is necessary [32]. Using various tremor features in the frequency and time domain (amplitude, power distribution, frequency dispersion, median frequency, etc.), machine learning (ML)-based approaches were developed to differentiate the typical human daily activities and estimate tremor severity during the activity [15,17,33]. ML applies mathematical algorithms that can discover subjective designs or structure in the data and predict the result for the new inputs based on the experienced models [34,35].

Despite the evidence suggesting ML’s efficiency for identifying fatigue-induced tremor, the application to T1DM is limited to our preliminary work. In pursuit of a solution to predict hypoglycemic events via the detection of hand tremors, the goal of this paper is to document an ML-based algorithm to recognize and classify voluntary effort and fatigue phases. The algorithm was built using data from a maximum voluntary contraction test, and time and frequency domain features were extracted. The accuracy of detecting voluntary movement (rest and effort tasks) and fatigue phases (early and late) are presented.

## 2. Background

### 2.1. Machine Learning and Tremor Recognition

Recognition of rest, posture, and kinetic tremors are crucial for the accurate diagnosis and efficient treatment regime [17,24,36]. Several recent ML techniques have shown promise in diagnosing and classifying tremor-based diseases, monitoring the progression of the disease, and for estimating the treatment regime [17,24]. Achievements in machine learning algorithms and wearable sensor technology have the potential to address these challenges by enabling the remote analysis of movement disorders [34,37,38]. For example, applying a tree-bagged classifier, a tool that processes a smartphone’s accelerometer and gyroscope signals, showed promising results for remote evaluation of the Parkinson’s patients’ condition [39]. An automatic scoring system using ML algorithms such as decision tree (DT), support vector machine (SVM), discriminant analysis, random forest, and k-nearest neighbors (k-NN) for objective measurement and correct evaluation of Parkinsonian tremor severity was demonstrated in [40]. Also, ML approaches have used different classifiers and numerical analyses to distinguish patients with essential and PD tremors at the individual level [32,41,42]. In [22], the authors hypothesized that hand tremors are unique for each individual. They investigated the possibility of using the assumption for biometric identification. A leap motion device was used, and different feature extraction methods were applied to prove the concept with the classification precision rate of above 95%.

Using a Naive Bayesian algorithm with optimum features, an accuracy value of 97% for the distinguish between rest, posture, and kinetic tremors were reported in [25]. SVM model classification accuracies of 95.4% for determining tremor-like movements [43] and from 88.6% to 88.9% for recognizing resting tremors [44] were announced. In [17], accuracies of 92.3%, 92.1%, and 89.2% were reported for the prediction of rest, postural, and kinetic tremors using polynomial SVM, k-NN, and DT on the principal component analysis (PCA)-projected data, respectively.

### 2.2. Machine Learning and Fatigue Phases Recognition

Dobrea et al. [45] demonstrated an intelligent user interface (virtual joystick) based on a tremor signal capable of identifying tired and rest states. Using SVM, fatigue induced in the morning (physiological) was distinguished from the neuro-muscular fatigued state (combination between physiological and neuro-muscular or psychological) obtained in the afternoon as well as rest and fatigue states differentiated in the afternoon. In [7], PCA was performed to investigate the increase in physical and cognitive fatigue expressed as an enhancement of tremor amplitude and blinking that results in the error rate rise after an 8h implementation of light tasks with high precision. Finally, an ML system designed on heterogeneous ensemble-based voting classifier with an accuracy of 92% was employed to classify activities and fatigue states [29]. Effectiveness of the fatigue managing framework, with an average accuracy higher than 85%, was demonstrated when a random forest model comprised of less than seven features was tested [46]. Strohrmann et al. categorized the runners’ skill level and distinguished between three levels (beginner, intermediate, and advanced) with 76.92% accuracy applying SVM to analyze the accelerometer data collected from the upper body [47]. A recognition rate of 68% was obtained with k-NN clustering (k = 1) recording of the gait before and after exhaustion [48]. Linear regression and a parametric hidden Markov model with 0.83 ± 0.31 and 0.84 ± 0.43 root mean square errors, respectively, were utilized to evaluate fatigue that was measured from the kinematic changes in exercise performances [49].

Machine-learning techniques were also used to interpret the data and predict tremor without any earlier information: a time-sequence-based fuzzy support vector machine adaptive filter for tremor canceling was proposed [16]; Veluvolu et al. presented band-limited multiple Fourier linear combiner with Kalman filter and recursive least squares algorithms for recognizing the voluntary and involuntary motion from the accelerometer data in real time [23]; multistep prediction of physiological tremor in real time to reduce the time-varying delay applying a kernel-based learning technique with the average accuracy of 71 ± 1.89% was demonstrated in [35]. Yang et al. presented a teleoperation scheme based on SVM and variable gain control for simultaneously suppressing tremors and personifying control performance [19]. Finally, using a weighted frequency Fourier linear combiner and Kalman filter, the TREMOR Neurorobot screened the neuromusculoskeletal system to estimate the frequency and amplitude of both—voluntary and tremulous motions, and then excited upper limb muscles to compensate for the tremor [50].

## 3. Experimental Setup and Data Processing

### 3.1. Participants

A balanced group of 40 right-handed adults (19 males and 21 females, 20 healthy (mean age: 23.16 years, standard deviation (SD): 4.525; mean body mass index (BMI): 23.158, SD: 3.051) and 20 subjects with T1DM (mean age: 22.1 years, SD: 7.677; mean BMI: 24.585, SD: 4.841)) was recruited from the local community to participate in the study. The study protocol was approved by the Texas A&M University Institutional Review Board. Participants were informed of the experiment details and signed informed consent forms before the experiment began. The investigation was conducted by trained personnel; the participants’ state was observed and verbally encouraged. No blood sugar level data were collected before or during the study.

### 3.2. Procedure

**Figure 1:**
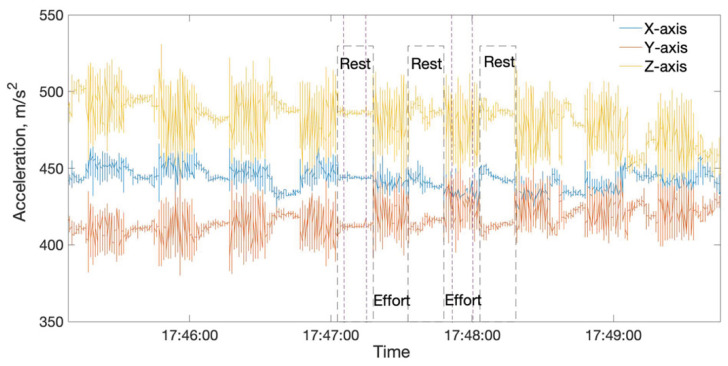
Effort and rest task data (middle 10 s of each event was taken for the analysis).

The experimental protocol was designed to extract and evaluate hand-tremor data from two stages (rest and effort) of isometric handgrip contractions. Participants were seated upright on the chair with their elbow flexed at 90° while resting the lower arm on an armrest. To execute of the effort task, they were asked to squeeze a 300 g hand dynamometer (BIOPAC, Goleta, CA, USA). The effort task started with measurement of the maximal handgrip strength implementing three isometric maximum voluntary contractions (MVCs) [51,52], 5 s each, with 60-s rest in between and continued with the enforcement of 30% MVC, calculated using the maximum amplitude of the MVCs. Participants were required to maintain the 30% MVC for 15 s, followed by 15 s of rest until exhaustion (Figure 1). Real-time feedback of the force exertion was displayed on a computer screen to ensure that participants sustained the handgrip force exertion at 30% MVC level as closely as they could. The experiment’s duration varied from 30 to 119 min as the study continued until the subject stated that he/she was tired. Each participant’s data were divided into three parts corresponding to the fatigue phases (early: first one-third, middle: middle one-third, and late: last one-third of the fatigue trials). We compared the time and frequency domain features (listed in the Data Processing Section) in early and late fatigue phases during the handgrip motor fatigue tasks to determine the fatigue onset.

### 3.3. Wearable Tremor Detection System

The hand tremor signal was recorded using a 3-axial accelerometer (ADXL 355, minimum full-scale range ±3 g) with an amplitude of the output voltage signal proportional to the corresponding axial acceleration. The data were collected from the wrist and middle finger of the participant’s dominant hand. Acceleration data were sampled at 45 Hz, digitized by 14-bit microcontroller Arduino Uno R3 (Adafruit), and transmitted to a computer. To exclude the components affected by body position changes, 3 Hz cut-off frequency contents were applied. To eliminate artifacts as external noises and aliasing, the dynamometer signal was low-pass-filtered using a first-order Butterworth filter at a cut-off frequency of 15 Hz. Only the middle 10 sec from each event were extracted for data analysis (Figure 1).

### 3.4. Data Processing

The collected data were segmented and summarized into a small set of features and later inputted into the machine-learning algorithm. To find the optimal period for calculating the features and duration of each participant’s tasks, the time-series signal was segmented into equal segments with two specific lengths of the sliding window (45 and 90 samples per window with duration 1 and 2 s, respectively). For the windows with lengths of 45 and 90 samples, the number of segments were 33,040 and 16,520, respectively. As the participants repeated rest and effort tasks until they were fatigued, the number of windows related to rest and effort tasks were equal. In a preliminary study, accuracy performances of 85.2–87.3% and 79.5–86.0% were achieved using amplitude peaks and time domain features, respectively. Considering the obtained results, features in the time and frequency domain were extracted for each segment of the accelerometer signal to investigate the tremor characteristics. In the time domain, root mean square (RMS) was first computed, and temporal features such as maximum, minimum, and mean amplitude, number of peaks above it, signal magnitude vector, standard deviation, and Pearson correlation between the axis *x*, *y*, and *z*, were extracted. The mean amplitude was calculated by averaging peak-to-peak amplitudes in the RMS signal [17,40]. The distribution of power of the input signal at various frequencies was estimated by power spectral density (PSD). In the frequency domain, spectral power and number of peak frequencies (PF) for each window were computed. PF was computed as the frequency at maximum power in the averaged spectrum. Eighty-two features were extracted for the data collected from the wrist and finger sensor (41 features each), as follows: RMS, min, max, mean, and number of peaks above the mean were calculated for three axes and signal magnitude vector (20 features); Pearson correlation coefficients (3 features); PSD and number of peaks of the calculated signal magnitude vector (2 features): SDs of RMS, min, max, mean of three axes and signal magnitude vector (16 features).

To recognize and classify tasks (rest or effort in addition to early and late fatigue), four classification algorithms were explored, namely DT, SVM, k-NN, and ensemble classifier (EC). The fine tree classifier type of decision tree algorithms with many leaves (up to 100 splits) were used to refine classes’ distinctions. Three kernels (cubic, medium, and fine gaussian) were performed when SVM was implemented. For the medium and fine gaussian SVM, kernel scales of the divergences between classes were set to sqrt(P) and sqrt(P)/4, respectively (where P is the number of predictors). Three k-NN classifiers (weight, cosine, and fine) with a number of neighbors set to 1 were used. In the weight and cosine k-NN, medium distinctions between classes based on distance, weight, and cosine distance metrics were applied. From the group of the ensemble classifiers, bagged trees (BT) based on the subspace k-NN and random subspace (RS), based on random forest with decision tree learners, were chosen. Data analysis was processed using MATLAB software (release 2019b; The MathWorks, Natick, MA, USA) [53].

An analysis was implemented on training data and validated on test data to estimate the classifiers’ performance. Data were split into two parts: 80% from data were used to train the classifier’s model, and 20% were used as test data to validate it. Evaluation of the classifier’s performance was assessed based on various metrics using 5-fold cross-validation. Accuracy (Equation (1)) can be defined as the rate of accurately classified events among the tested ones [54]. The classifier’s ability to recognize the effort tasks and fatigue phases were estimated computing the parameters recall, specificity, precision (PPV), and F1-score, which can be thought of as a measure of true positive rate (classifier completeness), true negative rate, positive predicted values (classifiers exactness) and balance between the classifiers completeness and exactness through, respectively, Equations (2)–(5) [54].

(1) $$\mathrm{Accuracy}=\frac{\mathrm{TP}+\mathrm{TN}}{\mathrm{TP}+\mathrm{FP}+\mathrm{TN}+\mathrm{FN}}\times 100\;[\%]$$

(2) $$\mathrm{Recall}=\frac{\mathrm{TP}}{\mathrm{TP}+\mathrm{FN}}\times 100\;[\%],$$

(3) $$\mathrm{Specificity}=\frac{\mathrm{TN}}{\mathrm{FP}+\mathrm{TN}}\times 100\;\left[\%\right],$$

(4) $$\mathrm{Precision}=\mathrm{PPV}=\frac{\mathrm{TP}}{\mathrm{TP}+\mathrm{FP}}\times 100\;\left[\%\right],$$

(5) $$F1-\mathrm{score}=2\frac{\mathrm{PrecisionxRecall}}{\mathrm{Precision}+\mathrm{Recall}}\times 100\;[\%],$$

where TP, TN, FP, and FN refer to true positive, true negative, false positive, and false-negative rates, respectively; TP: the task is labeled as rest/early fatigue and input is predicted as rest/early fatigue, respectively; TN: the task is labeled as effort/late fatigue and input is predicted as effort/late fatigue, respectively; FP: the task is labeled as effort/late fatigue and input is predicted as rest/early fatigue, respectively; FN: the task is labeled as rest/early fatigue and input is predicted as effort/late fatigue, respectively.

To organize classifiers and visualize their performance, receiver operating characteristic (ROC) curves were plotted, and area under the curves (AUCs) was calculated to assess the performance of the classification rules.

## 4. Results

### 4.1. Rest and Effort Recognition

**Figure 2:**
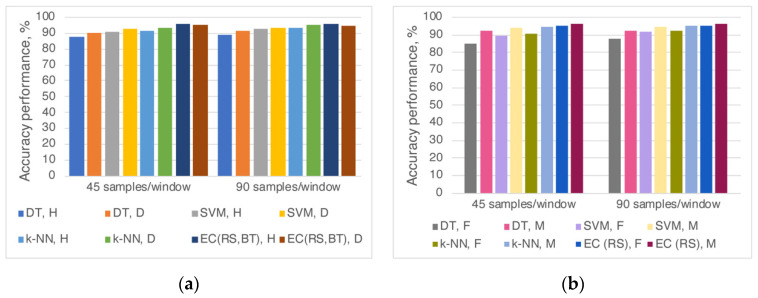
Accuracy performances of the algorithms for rest and effort events classification when participants were divided according to (**a**) health condition (H—healthy; D—diabetes) or (**b**) gender (F—female; M—male).

Four machine learning algorithms (DT, SVM, k-NN, and EC) and window sizes of 45 and 90 samples were applied to recognize and classify the rest and effort (30% MVC) events. The performances of the algorithms to distinguish rest and effort activities are summarized in Table 1. Differences between algorithms for healthy and T1DM samples are presented in Figure 2. As shown in Table 1, when healthy and diabetic patients were combined, the accuracy performance varied between 90.0% and 96.1% depending on the algorithm and window size used. The accuracy slightly increased (less than 1%) depending on segmentation. For non-parametric algorithms (DT, SVM, and k-NN), the size of 90 samples/window improved the accuracy compared to 45 samples/window. In contrast, for the ensemble classifier with a random subspace k-NN (EC (RS)), 45 samples/window showed better performance. The highest precision (96.1%) was obtained when the EC (RS) method with the segmentation of 45 samples/window was applied.

When performances of the algorithms were compared based on health conditions (healthy and T1DM participants), the accuracy was up to 3% higher for the diabetes patients when DT, k-NN, and SVM analyses were performed (Figure 2a) regardless of sampling/window size. Using EC and window size 45 samples, the accuracy values were similar (95.6% vs. 95.5% for healthy and diabetes, respectively). However, for window size 90 samples, the performance was 1.2% better for healthy (95.9%) than diabetic patients (94.7%).

When participants were separated according to gender, the performance accuracy values varied between 85.5% and 96.6%, depending on the window size and classification methods that recognized the voluntary effort (Figure 2b). The gender effect on model performance was visible when non-parametric classifiers (DT, SVM, and k-NN) were applied; rest and effort tasks were distinguished with up to 6% higher accuracy in males. On the other hand, using EC (RS) that achieved the best accuracy, the model’s efficiency in males was 1% higher.

### 4.2. Early and Late Fatigue Recognition

The study’s second objective was to detect the fatigue onset comparing the tremor features in early and late fatigue phases during the handgrip motor fatigue experiment. After recognizing the voluntary effort from the collected data, we trained and tested the machine learning algorithms to classify early and late fatigue phases with the lengths of the windows at 45 and 90 samples.

Accuracy performances of the algorithms used that classify early and late fatigue phases are presented in Table 2. Appling ensemble classifier based on random subspace k-NN, the fatigue stages were distinguished with an accuracy of ~98% for both window lengths. The fatigue phases’ poorest recognition was assessed by DT classifier (84.1% and 83.1%). Similar to the models that recognize the effort tasks, DT algorithm identified the fatigue phases with almost 14% lower efficiency than EC (RS). Non-parametric models accomplished accuracy performance differences of up to 2.7% when compare both window sizes. SVM recognized the fatigue phases with an accuracy of 94.5% and 91.8% for 45 and 90 samples/window, respectively. Ensemble classifier achieved almost the same performance for 45 and 90 samples/window (97.8% and 97.9%, respectively).

**Figure 3:**
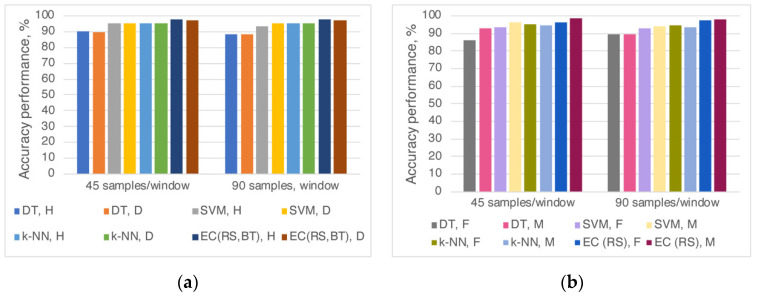
Accuracy performances of the algorithms for early and late fatigue classification when participants were divided according to (**a**) health condition (H—healthy; D—diabetes) or (**b**) gender (F—female; M—male).

Figure 3a represents the efficiency of the applied algorithms when the participants were separated according to the health condition. The most accurate model for the fatigue phase’s recognition was EC (RS) with values of the accuracy performance of 97.2% (diabetes patients; 45 and 90 samples/window), 97.8% (healthy participants; 90 samples/window), and 97.9% (healthy participants: 45 samples/window).

When the participants were separated according to gender, the early and late fatigue phases with the highest accuracy were in the male group when the EC (RS) was applied (Figure 3b). For this group, 98.4% efficiency for the classification of the fatigue phases was achieved with a windowing of 45 samples. The accuracy reached a value of 96.5% for the female group. When applying k-NN algorithm for both window lengths, the fatigue stages recognition effectiveness was higher in females (95.3% and 94.8%) compared to those of males (94.9% and 93.7%).

Evaluation metrics of the classification models’ ability were computed; the results are presented in Table 3.

The accuracy performance values of algorithms using the validation dataset were similar to the results obtained when the test data was used. The best accuracy performance (98.04%) was achieved by applying EC in males with 90 samples/window. The highest number of misclassified events was when DT model for both window lengths was used.

Recall and specificity (Equations (2) and (3)) are defined as the proportion of correctly identified positive cases (early fatigue phases) and the proportion of negative cases (late fatigue phases), respectively. When EC was applied, the highest ratio of the correctly identified early (between 94.24% and 98.95%) and late (between 96.65% and 97.60%) fatigue phases were reached. For DT model, correctly classified samples’ ratio varied between 83.72% and 94.31% for early fatigue and between 73.69% and 89.80% for late fatigue, depending on the separation and window lengths.

The precision (Equation (4)) of the classifier was estimated using the proportion of correctly detected early fatigue phases and the total classified early fatigue phases; it relates to the low false late fatigue events rate. The highest precision values (~97%) were achieved using EC (Table 3).

The weighted average of precision and recall-F1 Score (Equation (5)), that takes into account the false positives (late fatigue phase classified as early) and false negatives (early fatigue phase classified as late) is an overall measure of a model’s accuracy. The EC’s satisfactory performance was confirmed by F1 Score values between 97% and 98% (Table 3).

**Figure 4:**
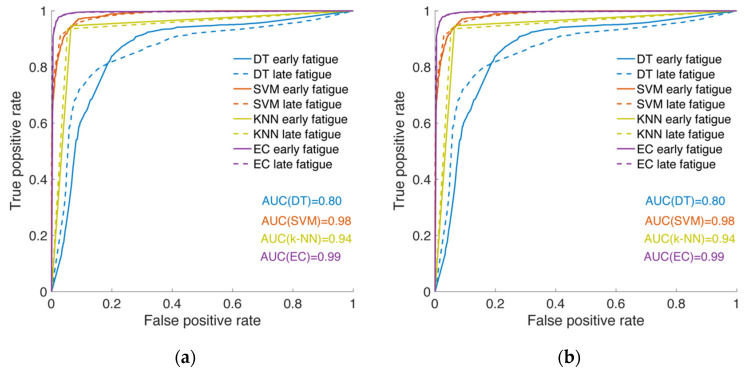
Receiver operating characteristic (ROC) curves of the trained models with (**a**) 45 and (**b**) 90 samples/window; early fatigue phase is plotted with straight line, late fatigue phase—with dashed line.

The classification ability of the models was estimated by plotting ROC curves (Figure 4). ROC graphs illustrate correctly classified early fatigue phases (TP rate) against correctly classified late fatigue phases (TN rate) over a range of algorithm confidence thresholds. Excellent results with very few misclassified events were shown by employing EC for both window lengths. The fatigue phases were classified with approximately the same accuracy when EC (45 and 90 samples/window), SVM, and k-NN models (45 samples/window) were applied (Figure 4). The least effective classification ability was demonstrated by the DT algorithm with differences in probability to recognize both fatigue phases.

## 5. Discussion

### 5.1. Rest and Effort Recognition

We estimated the performance of the classifiers that recognized voluntary effort while participants implemented the submaximal handgrip fatigue task. A comparison between the proposed and existing algorithms reported in Section 2 demonstrates the proposed one’s better accuracy performance for rest and kinetic tremor recognition (Table 4). The most accurate proposed model that achieved 96.1% effectiveness of voluntary effort recognition was the ensemble classifier with random subspace k-NN and a window length of 45 samples (Table 1). The reached accuracy is less than 1% lower than the reported in [25] accuracy of 97% (Naïve Bayesian algorithm), Table 4. Even though both algorithms detected and recognized rest and action tasks implemented by participants suffering from chronic diseases, several key differences between these studies such as the groups examined (Parkinson’s disease participants vs. healthy and T1DM participants), implemented tasks (index-nose task vs. many time repeated effort task squeezing a 300 g dynamometer) and duration of the experiment (2 min vs. average experiment duration of 60.625 min, SD 18.968) makes the comparison difficult. In addition, the reported accuracy of 97% in [25] was calculated based on 201 windows of test data (each with a duration of 4 s), while in our study we estimated models’ performance based on 6608 windows with a duration of 1 s and 3304 windows with 2 s. In both studies, accelerometer data were collected.

Of the non-parametric models used, k-NN algorithm classified the fatigue phases with the highest accuracy (94.2%). k-NN uses the distance from the unknown observation to the training samples and the number of the nearest neighbors [55,56]. Various approaches were included to improve the algorithm performance—taking multiple distance metrics to develop various ensemble members, measuring distances using genetic algorithms, and using random subspaces to generate component k-NN classifiers [56,57]. Applying k-NN, lower accuracies of 87% (accelerometer and gyroscope data) and 92.1% (accelerometer data) were achieved in [25] and [44], respectively (Table 4). In the case when the SVM method was accomplished, we obtained 93.3% classifier effectiveness (Table 1), unlike 92%, 70%, and 89% accuracies values for the rest tremor recognition (with feature selection utilized) that were reported in [17], [25], and [44], respectively (Table 4). With the DT algorithm applied, the proposed model’s accuracy performances and related works were around 90% (Table 4).

When the participants were separated based on gender (Figure 2b), the difference in performance accuracy was around 1%, applying EC (RS). In the case when the sliding window is 45 (90) samples, voluntary movements were recognized as follows: all participants—96.1 (95.5)%, females—95.4 (95.4)%, males—96.6 (96.5)%. It appears that this model classified events according to different tremor characteristics’ trends based on gender. Various studies imply that gender can influence hand tremors; a unanimous opinion still has not been established yet. The following was observed: men achieved higher scores and experienced more severe hand tremors during the effort task [58,59]. A significant distinction in 10 Hz tremor amplitude in males has been found compared to females, but with no difference in tremor peak frequencies [60]. There were slightly lower tremor frequencies in women according to [61] or contrariwise, according to [62]: lower frequencies with slightly higher amplitude values were observed in men based on the effect that women have significantly smaller hands compared to men.

The accuracy obtained with EC (RS) for 45 and 90 samples/window was close: 96.6% and 96.5% for males (Figure 2b). For females, the accuracy was not dependent on window length when EC (RS) was used because 95.4% value was observed for both window lengths. In this group, the accuracy performance of distinct models was the most noticeable, changing from 85.9% (DT, 45 samples/window) to 95.4% (EC (RS), 45 samples/window). The window length did not significantly affect the non-parametric models’ accuracy in males because the differences between 45 and 90 samples/window were less than 1%. On the other hand, there were up to 3% with the same parameters in the female group.

### 5.2. Early and Late Fatigue Recognition

The algorithms’ accuracy performance for the estimation of fatigue-induced tremor characteristics and detection of early and late fatigue phases was evaluated. All proposed models demonstrated improved accuracy performance compared to the existing literature (Table 4).

The most effective classifier with ~98% was ensemble classifier based on the random subspace k-NN (Table 2 and Table 4). An accuracy of 92 % for detecting the fatigue stages was reported when the heterogeneous ensemble learning voting method using data subset prediction classes was employed [29] (Table 4). In [46], the fatigue managing framework’s effectiveness with an average accuracy of 89.7% and 87.9% (depending on the performed tasks) when a random forest model comprising less than seven features was tested. Applying the SVM algorithm, fatigue recognition with lower accuracies (78.7% and 82.0%, depending on the performed tasks [46]) compared to the performance of the proposed model (94.39% and 93.06%, depending on the sampling window, Table 4) were demonstrated as well.

Using a k-NN algorithm, we reached higher effectiveness (96% and 94.23% in case of 45 samples/window and 90 samples/window, respectively, Table 4) compared to the preliminary results that demonstrated k-mean clustering performance of 31.57% correct identification of tired state and 85.71% of the rest state reported in [45].

The separation of participants according to a health condition (Table 3) mostly affected the DT model as it increased the exactness of fatigue phase classification from 79.89% and 82.52% to 89.73% and 87.73% (45 and 90 samples/window, respectively) for healthy participants and to 88.40% and 91.24% (45 and 90 samples/window, respectively) for the diabetes participants. The accuracy increased by up to 2% with the SVM model, while the accuracy changed slightly with the EC (RS) and k-NN models applied. The comparison of these models’ effectiveness shows a weak influence of the window size. For the non-parametric algorithms, the accuracy performance when the participants were separated according to health condition is higher (87.73–95.86%) than in the case where all participants were combined (79.89–94.5%) except for k-NN model applied for healthy subjects. However, health condition does not influence the accuracy performance of the ensemble classifier; the values are between 97.2% and 97.9% for both window sizes. The complex nature of fatigue and the factors affiliated among adults suffering from T1DM is the reason for the limited number of investigations of the influence of fatigue on this group of patients [63] as well as the lack of papers investigating machine-learning classifications based on fatigue-induced tremors in healthy and T1DM participants. MVC amplitude significantly decreases, and fatigue onset occurs sooner in T1DM participants compared to the control group according to [31]. They also reported that peripheral neuropathy caused by T1DM reduces muscle activation and greater fatigability due to decreased nerve condition velocity. The multidimensional nature of fatigue confirms the difficulties of distinguishing both groups based on the proposed models.

The gender grouping of participants increased the accuracy performance of the DT model from 73.89% and 82.52% to 89.70% and 87.31% (45 and 90 samples/window, respectively) in females and to 91.8% and 89.22% (45 and 90 samples/window, respectively) in males (Table 3). For the EC (RS) model, accuracy performances were higher in females (97.76% and 97.66% for 45 and 90 samples/window, respectively) compared to when participants were not gender-grouped. In males, accuracy performance was higher (98.04%) for 90 samples/window and lower (97.36%) for 45 samples/window compared to the no grouped participants (Table 3). The effect of gender on fatigue was the object of various studies with no confirmed conclusion regarding the causes of its appearance and performance. The accuracy of the proposed EC (RS) model in males recognizes fatigue phases with higher accuracy than in females. One possible reason for this could be higher tremor features in men, greater fatigue resistance, and superior females’ hand stability [60,64], or hand volume [62].

In terms of evaluation metrics, when participants’ data were not divided according to a health condition or gender, recall and specificity metrics for fatigue phase recognition were almost identical (a difference of less than 1%) using k-NN and EC (RS) classifiers (i.e., early and late fatigue phases were recognized with nearly the same accuracy (Table 3)). The data were well-balanced; the number of events in both classes was very similar because the first and last third of the recorded data for each participant were used. When DT or SVM was applied, the number of misclassified samples increased, and the prediction efficiency decreased. These trends were also observed when the participants were separated into two groups: healthy–diabetes or males–females.

## 6. Conclusions

In this study, we designed an experimental protocol to collect large number of test sets to extract and evaluate hand-tremor data from two-stages (rest and effort) of isometric handgrip contractions. A balanced group consisting of healthy and T1DM patients was recruited. Even though there are similar studies, replication using a different population and tasks makes a good contribution for validation purposes. A system that identifies and classifies voluntary effort and fatigue phases was developed. To find the optimal period for calculating the features and duration of each participant’s tasks, the accelerometer data were segmented into windows with different lengths (45 and 90 samples/window), and time and frequency domain features were extracted. Machine-learning techniques (decision tree, k-nearest neighbors, support vector machine with different kernels, and ensemble classifiers) were applied for rest/effort tasks and early/late fatigue phase detection. The algorithm recognized, labeled and evaluated the tremor data during rest and effort tasks with improved accuracy compared to the existing models. The recognition of rest and effort tasks using the ensemble classifier based on the subspace k-NN and segments’ lengths 45 and 90 samples/window were deemed to be the most accurate (96.1%). The highest accuracy (~98%) of early and late fatigue stages detection was achieved using the same classifier and window length. The effect of fatigue on physiological tremor in groups separated by health condition and gender was investigated. It is established that health condition does not influence the accuracy performance of ensemble classifier; the values were between 97.2% and 97.9% for both window sizes. We found that proposed model is not affected by gender and identifies effort tasks with the same accuracy, while the fatigue phases were recognized with higher accuracy in males than in females.

It is essential that the following limitations are addressed in future work. Because blood glucose levels can affect fatigue in adults suffering from T1DM [12,31], the information’s absence is the main constraint of the study. The results can be affected by diabetes-related complications, for which data were not collected. Another limitation is that finger tremors can be affected by the hand’s posture, gripping and squeezing of the hand, and dynamometer [7,62]. Experimental protocols should also be extended, including different fatiguing tasks. Although an accelerometer can be effectively used to capture hand tremor changes in static activity, it does not assess the effect of muscle fatigue during the implementation of dynamic activities that requires measurement of surface electromyography [18]. To minimize the equipment constraints, accelerometer data could be combined with data collected by a gyroscope.

## Figures and Tables

**Table 1:** Accuracy performance of the algorithms classifying rest and effort events *.

Algorithm	Samples/Window	
	45	90
DT	90.0% (fine)	90.5% (fine)
SVM	92.4% (fine)	93.3% (cubic)
k-NN	93.2% (weight)	94.2% (weight)
EC	96.1% (subspace)	95.5% (subspace)

* All participants combined.

**Table 2:** Accuracy performance of the algorithms classifying early and late fatigue phases *.

Algorithm	Samples/Window	
	45	90
DT	84.1% (fine)	83.1% (fine)
SVM	94.5% (fine Gaussian)	91.8% (fine Gaussian)
k-NN	96.0% (weight)	94.2% (fine)
EC	97.8% (subspace)	97.9% (subspace)

* All participants combined.

**Table 3:** Evaluation metrics of the classifiers’ performance.

	**45 Samples/Window**					**90 Samples/Window**				
	**Accuracy, %**	**Recall, %**	**Spec., %**	**PPV, %**	**F1-Score, %**	**Accuracy, %**	**Recall, %**	**Spec., %**	**PPV, %**	**F1-Score, %**
**All participants**										
DT	79.89	83.72	76.08	77.71	80.61	82.52	91.35	73.69	77.64	83.94
SVM	94.39	90.65	98.12	97.96	94.16	93.06	89.37	96.75	96.50	92.80
k-NN	96.00	96.85	95.16	95.23	96.03	94.23	94.41	94.05	94.08	94.24
EC	97.71	98.02	97.40	97.41	97.71	97.39	97.66	97.12	97.13	97.40
**Healthy participants**										
DT	89.73	93.32	86.16	87.02	90.06	87.73	87.36	88.10	88.01	87.69
SVM	95.47	96.47	94.46	94.55	95.50	96.10	97.02	95.17	95.26	96.13
k-NN	95.37	96.47	94.28	94.37	95.41	94.05	94.80	93.31	93.41	94.10
EC	97.39	98.14	97.60	97.60	97.87	97.40	98.14	96.65	96.70	97.42
**Diabetes patients**										
DT	88.40	87.61	89.20	89.01	88.30	91.24	92.66	89.82	90.14	91.38
SVM	95.86	96.16	95.47	95.49	95.83	95.10	96.15	94.04	94.18	95.16
k-NN	95.12	94.94	95.30	95.27	95.11	95.62	97.20	94.04	94.24	95.70
EC	95.82	94.24	97.39	97.30	95.74	97.90	98.95	96.84	96.92	97.92
**Males**										
DT	91.80	94.31	89.30	89.74	91.97	89.22	88.63	89.80	89.68	89.15
SVM	96.09	97.45	94.75	94.85	96.13	94.71	94.9	94.51	94.53	94.72
k-NN	95.61	97.25	93.97	94.12	95.66	95.10	96.47	93.73	93.89	95.16
EC	97.36	97.65	97.08	97.08	97.36	98.04	98.82	97.25	97.30	98.05
**Females**										
DT	89.70	91.35	88.06	88.41	89.85	87.31	87.67	86.96	87.09	87.38
SVM	93.85	90.68	97.01	96.80	93.64	93.49	94.67	92.31	92.51	93.57
k-NN	94.85	94.51	95.19	95.14	94.82	96.49	95.33	97.66	97.61	96.46
EC	97.76	98.67	96.85	96.90	97.77	97.66	98.00	97.32	97.35	97.67

**Table 4:** Effectiveness of the proposed model and existing algorithms.

**ML** **Algorithm**	**Recognition**	**Sensor**	**Accuracy Performance**	**Reference**
**Voluntary effort recognition**				
DT, SVM, k-NN, EC	Rest and effort events	Accelerometer	90.0–96.1%	Table 1
Naïve Bayesian k-NN SVM ANN	Rest, posture, and kinetic tremor	Accelerometer	97% 87% 70% 91%	[25]
SVM	Rest tremor	Accelerometer+ Gyroscope	88.6–88.9%	[44]
SVM DT k-NN DT	Rest tremor Rest tremor with mental stress Postural tremor Intention tremor	Accelerometer+ Gyroscope	92.3% 86.2% 92.1% 89.2%	[17]
**Fatigue stages recognition**				
DT, SVM, k-NN, EC	Early and late fatigue phases	Accelerometer	79.89–97.71%	Table 2
Heterogenous EC	Fatigue stages	Accelerometer+ Gyroscope	92%	[29]
Random forest SVM	Fatigue managing framework	Accelerometer+ Gyroscope	89.7%; 87.9% 78.7%; 82%	[46]
SVM	Tired state Rest state	3D-sensing device	31.57% 85.71%	[45]
